# 

**Published:** 1918-04-13

**Authors:** 


RATES OF SUBSCRIPTION: Twelve months, 6/6; Six months, 4/-; three months, 2/-, post free to any address in the United Kingdom. Abroad, Twelve months, 8/8; Six months, 5/-; Three months, 2/6, post free. **THE HOSPITAL "Index" to Volume LXII. April 1917 to September 1917, is now ready, price 2½d. per copy, post free.** Address The Manager, THE HOSPITAL, "The Hospital" Building, 28/29 Southampton Street, Strand, London, W.C. 2.

28 & 29 SOUTHAMPTON STREET, STRAND, LONDON, W.C.

### Gifts and Bequests.

A number of bequests have been made to Liverpool institutions under the will of the late Mrs. S. A. Barrow. Amongst them are £300 each to the Liverpool School for the Indigent Blind and the Liverpool Home for Incurables, and £200 each to the Royal Infirmary, the Northern Hospital, the Stanley Hospital, the Southern Hospital, the Bootle Borough Hospital, and the Children's Infirmary.

At a meeting at the Royal Infirmary, at Edinburgh, it was stated that Miss H. M. Murray, formerly of that city, had left about £60,000 to found a home near Edinburgh for wounded and invalided sailors and soldiers.

Mr. Stanley Baldwin, M.P., Joint Financial Secretary to the Treasury, has given £5,000 to the Kidderminster Infirmary, to be used in the erection of a children's hospital for the borough.

King Edward's Hospital Fund will benefit by a legacy of part of the residue of the estate of Miss M. A. Fitze, of Hampstead.

The ultimate residue of the estate of Miss Louisa Richards, of Barnsbury, who left £16,488, is to be divided equally between King's College Hospital, the Salvation Army, and Dr. Barnardo's Homes.

Miss Mary Tufnell, of Beccles, has left £50 each to Beccles Hospital, the Brighton and Hove Dispensary, and Sussex County Hospital.

The Manchester White Heather Society raised £10,176 last year for the British Red Cross Society.

The British Committee of the French Red Cross has received a further donation of £4,000 from Lady Galway on behalf of the French Colony in Adelaide.

Mr. Middleton Jameson has sent a donation of £2,000 to University College Hospital in memory of his brother, Sir Starr Jameson, who received his medical education at the hospital, and was a member of the committee at the time of his death.

Lieutenant C. S. Haslam, of Helperby, Yorkshire, has left £2,000 to the Children's Hospital to endow two cots.

The Chelsea Hospital for Women has received from the Trustees of the Zunz Bequest a donation of £1,000 towards clearing off the debt of £14,000 on the new building.

Mr. James Thompson, solicitor, of Hatfield, a director of the Union Assurance Company, bequeathed £1,000 to St. Bartholomew's Hospital and £100 to Potter's Bar Cottage Hospital.

### Matrons' and Sisters' Appointments.

Borough Hospital, Birkenhead.—Miss M. Carlin has been appointed second assistant matron. She was trained at the Sheffield Royal Hospital, where she was later sister. She has also been military sister at the East Suffolk Hospital, Ipswich, at the Royal Surrey County Hospital, and pupil housekeeper at the Norfolk and Norwich Hospital.

Bridgend and Cowbridge. Union Infirmary.—Miss Ellen Handley has been appointed superintendent nurse. She was trained at the Birmingham Union Infirmary; has been night nurse at Crossley Sanatorium, Kingswood; maternity and ward sister at Southampton Union Infirmary; night sister at Chesterfield Union Infirmary; and superintendent nurse at Banbury Union Infirmary.

City of Lincoln Infectious Diseases Hospital and Sanatorium.—Miss Eileen O'Kane has been appointed matron. She was trained at the Belfast Infirmary and Fever Hospital, has been charge nurse at and later assistant matron of the Newcastle Infectious Diseases Hospital, sister in charge of the Nurses' Home, Newcastle City Hospital, and matron of the Sunderland Borough Sanatorium.

City of Westminster Union Infirmary, Fulham Road.—Miss L. Collins, whose appointment as assistant matron was announced last week, was trained at Northampton General Hospital, and not at Darlington General Hospital, as was stated in the official particulars.

Dorset County Asylum, Herrison, Dorchester.—Miss Sarah Heron has been appointed matron. She was trained at Bolton Infirmary and Dispensary and at Bristol Fever Hospital, has been mental nurse at Cumberland Asylum, Garlands, Carlisle, and assistant matron at Stirling District Asylum.

Essex County Hospital, Colchester.—Miss E. Smith and Miss M. Baily have been appointed sisters. Miss Smith was trained at the Nottingham General Hospital, and has been sister at the Bradford War Hospital, the Royal Surrey County Hospital, and home sister at Cuddington Hospital, Epsom. Miss Baily was trained at the Worcester General Infirmary, and has been sister at the Royal Gwent Hospital, Newport, and at the Stratford-on-Avon General and Auxiliary Military Hospital.

Manor Court Military Hospital, Folkestone.—Miss Lavinia Esther Millen has been appointed sister. She was trained at the Royal Victoria Hospital, Folkestone, and has been sister at the Liverpool Merchants' Hospital, France.

Red Cross Hospital, Perth.—Miss Katherine Edith Ogilvy has been appointed sister. She was trained at Retford Hospital and Dispensary, later being night sister there. She has also been sister-in-charge of the Military Hospital, Broomborough.

Royal Portsmouth Hospital.—Miss E. M. Yeadon has been appointed night sister. She was trained at the above-named hospital, has been ward sister at the Kent and Canterbury Hospital, and ward and night sister at the County Hospital, Lincoln. She has also done private nursing.

**Note.—Particulars of Appointments for insertion in this column will be welcomed.**

### St. Peter's Hospital, Covent Garden.

Nearly £1,000 has been subscribed towards the £10,000 for which an appeal is being made by Mr. F. A. Bevan (the treasurer), Mr. Edwin Fox (the chairman), Dr. F. Swinford Edwards, and Dr. Peter Freyer on behalf of St. Peter's Hospital, Henrietta Street, Covent Garden, " to meet its pressing liabilities and assure the continuance of its work, which must otherwise be seriously curtailed or even stopped altogether."

### The Book Market.

LIST OF NEW BOOKS RECEIVED FROM THE FOLLOWING PUBLISHERS :—

Adam and Charles Black : "The Edinburgh School of Surgery before Lister." By Alex Miles. 5s. net.
"Radiography and Radio-Therapeutics." By Robert Knox. Vol. II. 15s. net.

Chatto and Windus : "Herbert Fry's Royal Guide to the London Charities," 1918. 1s. 6d.

Poor-Law Publishers, Limited : "Practical Surgical Nursing for Probationers." By C. Seymour Yapp. 3s. 6d. net.

Henry Frowde and Hodder and Stoughton : "Burns and their Treatment." By J. M. H. Macleod. 6s. net.

University of London Press : "The Treatment of Fractures." Vols. I and II. By R. Leriche. Edited by F. F. Burghard, C.B., M.S., F.R.C.S. 6s. net each.

Thacker, Spink and Co., Calcutta : "Diabetes : Its Causation and Treatment." By Lieut.-Colonel Waters, I.M.S. Rupees 4.

Scientific Press, Ltd. : "A B C of Treatment in Accidents and Illnesses." By S. M. Clarke. 1s. 3d. net. "Notes on Venereal Diseases for Nurses and Midwives." By Dr. Mary Scharlieb. 1s. 3d. net.

John Wright and Sons, Ltd. : "Fitting-out and Administration of a Naval Hospital Ship." By Edward Sutton. 8s. net.

### Annual Meetings.

Queen Charlotte's Lying-in Hospital, Marylebone Road, N.W.—On Tuesday, April 16, at 5 p.m., at the hospital.

